# Supplementary figures and images for: Multi-analyte liquid biopsies for molecular pathway guided personalized treatment selection in advanced refractory cancers: A clinical utility pilot study
Source: Front Oncol. 2022 Dec 23;12:972322. doi: 10.3389/fonc.2022.972322 (PMC9822573; doi:10.3389/fonc.2022.972322)

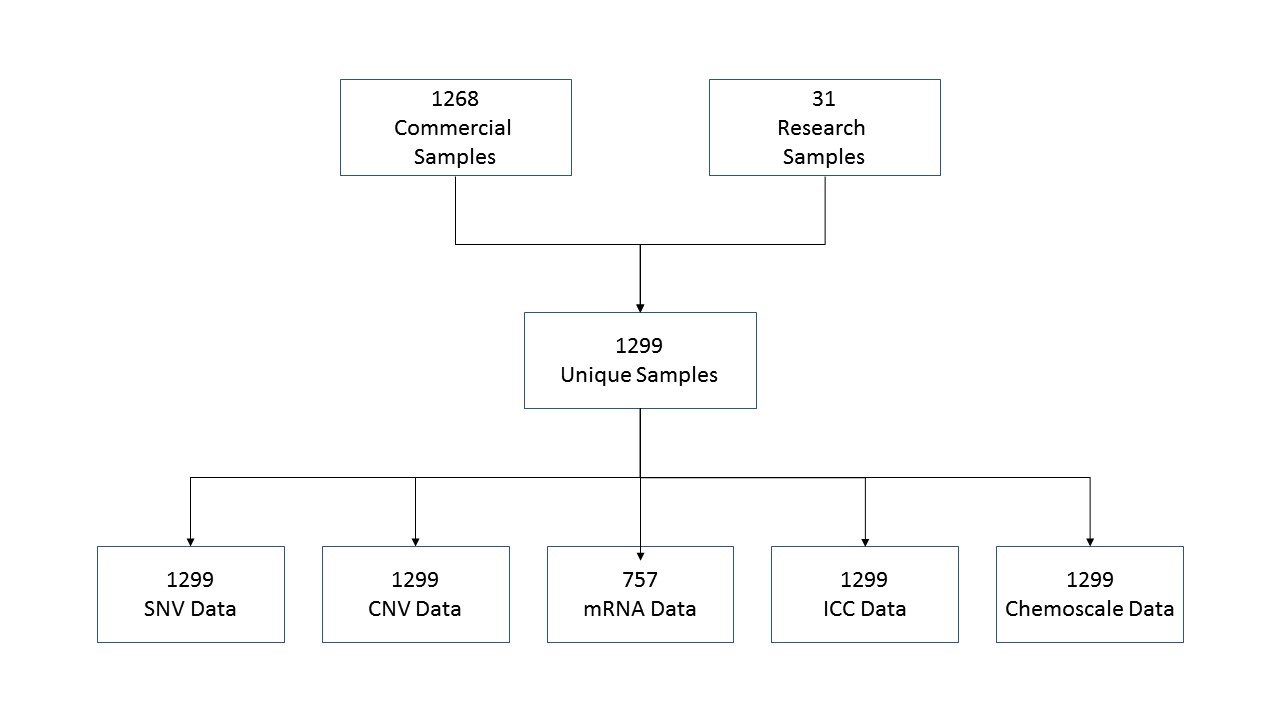

Supplement: Supplementary file 3 [file Image_1.jpeg]

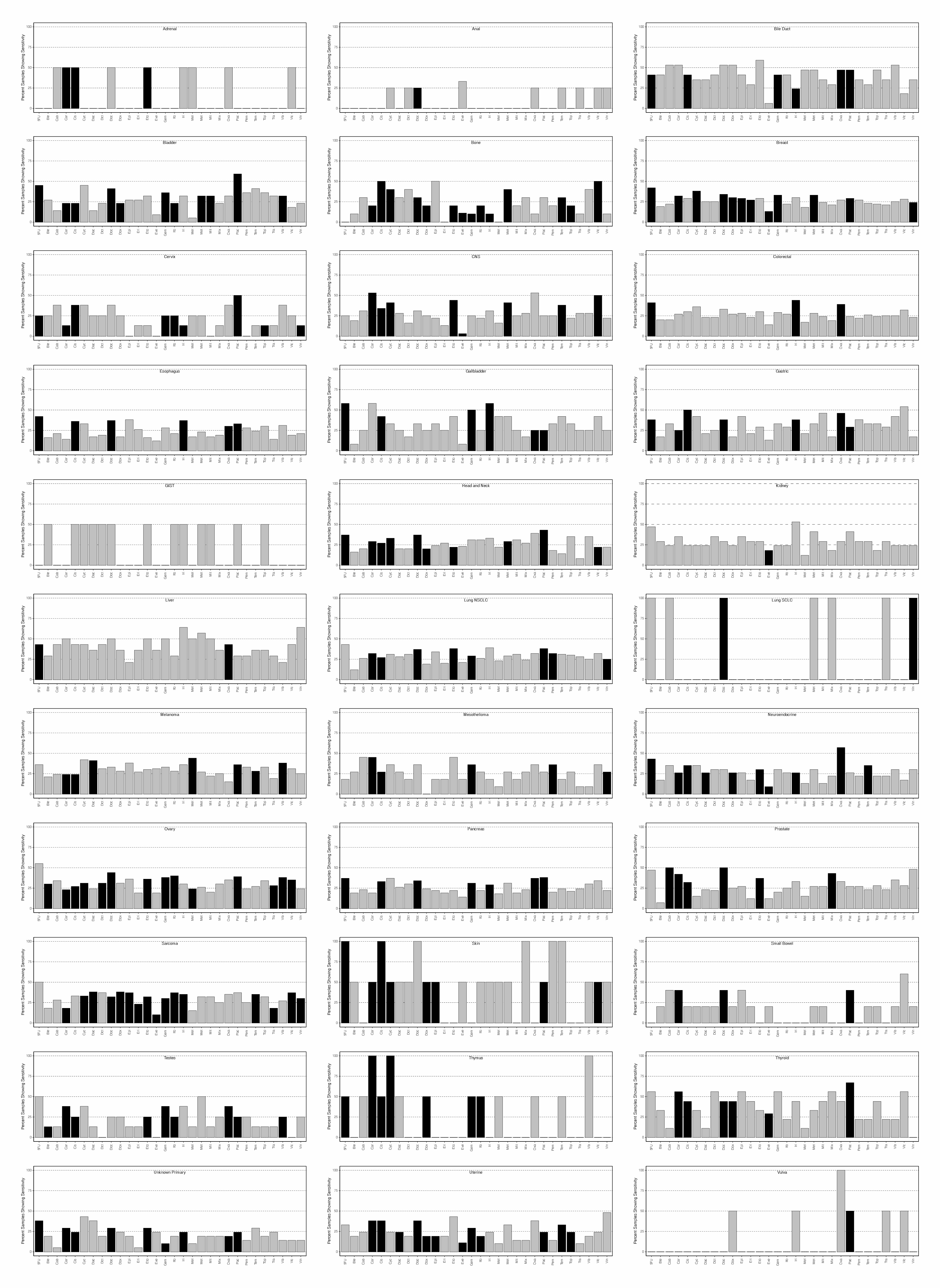

Supplement: Supplementary file 4 [file Image_2.jpeg]
